# CYP1B1 Augments the Mesenchymal, Claudin-Low, and Chemoresistant Phenotypes of Triple-Negative Breast Cancer Cells

**DOI:** 10.3390/ijms23179670

**Published:** 2022-08-26

**Authors:** Paul R. Hollis, Robert J. Mobley, Jyoti Bhuju, Amy N. Abell, Carrie Hayes Sutter, Thomas R. Sutter

**Affiliations:** Department of Biological Sciences, University of Memphis, Memphis, TN 38152, USA

**Keywords:** cytochrome P4501B1, targeted-therapy, claudin-low, survival, chemosensitivity

## Abstract

Cytochrome P4501B1 (CYP1B1) is elevated in breast cancer. Studies indicate a relationship between CYP1B1 and aggressive cancer phenotypes. Here, we report on in vitro studies in triple-negative breast cancer cell lines, where knockdown (KD) of CYP1B1 was used to determine the influence of its expression on invasive cell phenotypes. CYP1B1 KD in MDA-MB-231 cells resulted in the loss of mesenchymal morphology, altered expression of epithelial–mesenchymal genes, and increased claudin (CLDN) RNA and protein. CYP1B1 KD cells had increased cell-to-cell contact and paracellular barrier function, a reduced rate of cell proliferation, abrogation of migratory and invasive activity, and diminished spheroid formation. Analysis of clinical breast cancer tumor samples revealed an association between tumors exhibiting higher CYP1B1 RNA levels and diminished overall and disease-free survival. Tumor expression of CYP1B1 was inversely associated with CLDN7 expression, and CYP1B1^HI^/CLDN7^LOW^ identified patients with lower median survival. Cells with CYP1B1 KD had an enhanced chemosensitivity to paclitaxel, 5-fluorouracil, and cisplatin. Our findings that CYP1B1 KD can increase chemosensitivity points to therapeutic targeting of this enzyme. CYP1B1 inhibitors in combination with chemotherapeutic drugs may provide a novel targeted and effective approach to adjuvant or neoadjuvant therapy against certain forms of highly metastatic breast cancer.

## 1. Introduction

Cytochrome P450 1B1 (CYP1B1) belongs to the superfamily of mixed function oxidases involved in the cell detoxification and metabolic activation of many structurally diverse compounds [1,2]. CYP1B1 is a major extrahepatic enzyme involved in estrogen and xenobiotic metabolism [2,3]. In humans and mice, mutations of CYP1B1 lead to abnormal development of the trabecular meshwork and increased intraocular pressure that result in primary congenital glaucoma [4].

Breast cancer is a heterogeneous disease that is divided into subtypes based on the expression of the estrogen receptor (ER), progesterone receptor (PR), and amplification of the human epidermal growth factor receptor (HER2). Traditionally, five breast cancer intrinsic subtypes were commonly described including, normal breast-like, basal-like (BL), HER2-enriched (HER2^+^), luminal A (LumA) and luminal B (LumB) [5]. Each intrinsic subtype is its own distinct disease, displaying significant disparities in rate of incidence, therapeutic response, and survival [5]. Such classification has aided the development of targeted therapies for patients expressing ER, PR, or HER2. Moreover, further sub-classification, for example low-risk and high-risk LumA, has identified cohorts of patients more or less likely to benefit from specific treatment regimens [6]. In the case of patients with tumors lacking the expression of these receptors few targeted treatments are available. BL breast cancers, with minimal or undetectable ER and PR expression and are negative for HER2 amplification, are collectively termed triple-negative breast cancer (TNBC). TNBC tends to form large tumors that have a high rate of axillary lymph node involvement, and is more aggressive, accounting for 10–20% of invasive breast cancers [7,8]. These patients have a poor prognosis with low five-year and overall survival rates. Recently, an additional breast cancer subtype was identified by microarray gene expression analysis of breast tumors from humans and mice [9]. Named the Claudin (CLDN)-low (CL) subtype [9], CL tumors are mostly negative for ER, PR, and HER2 expression [10], and have diminished expression of epithelial tight junction, and cell-to-cell adhesion proteins including CLDN3, CLDN4, and CLDN7. CL tumors express markers associated with epithelial-mesenchymal transition (EMT) such as vimentin (VIM), and transcriptional repressors of cadherin 1 (CDH1) including twist family BHLH transcription factor 1 (TWIST1), twist family BHLH transcription factor 2 (TWIST2), zinc finger E-box binding homeobox 1 (ZEB1), zinc finger E-box binding homeobox 2 (ZEB2), snail family transcriptional repressor 1 (SNAI1) and snail family transcriptional repressor 2 (SNAI2) [5,8]. The identification and expression of features commonly associated with TNBC tumors suggest that the CL subtype is a subgroup of TNBC [11]. However, recent studies demonstrate that other intrinsic subtypes of breast cancer may express the claudin-low phenotype [12]. These reports indicate that CL may be a phenotype of multiple intrinsic breast tumor subtypes, albeit having a higher prevalence in the BL subtype [13,14].

Elevated levels of CYP1B1 are reported in many types of cancer. Breast cancer CYP1B1 elevation is reported for RNA and protein [15], suggesting that CYP1B1 may play a role in the initiation and/or progression of this cancer. Early studies of breast cancer cell lines report that aryl hydrocarbon receptor (AHR)-mediated induction of CYP1B1 is higher in cells having a mesenchymal morphology. Such ER-, invasive and tumorigenic cells lines have higher levels of CYP1B1 catalyzed estrogen 4-hydroxylase activity [16]. Two of these cell lines used here, the MDA-MB-231 and MDA-MB-157 lines, are classified by genome-wide transcript stratification as triple negative basal cells having a mesenchymal phenotype [17]. Subsequent studies further identify these cell lines as being of the CL subtype [5].

The functional significance of CYP1B1 expression in cancer was first demonstrated in endometrial cancers [18]. Immunohistochemical analysis demonstrate higher levels of CYP1B1 in endometrial adenocarcinoma relative to adjacent normal tissues. CYP1B1 protein is higher in undifferentiated and tumorigenic endometrial carcinoma cell lines. Depletion of CYP1B1 by small interfering RNA (siRNA) in these cells decreases cell proliferation, induces cell cycle arrest, increases apoptosis, and decreases Matrigel cell invasion [18]. To test the hypothesis that suppression of CYP1B1 diminishes the aggressive characteristics of TNBC cells, we used short hairpin RNA (shRNA) to lower CYP1B1 in MDA-MB-231 cells; to replicate certain findings, we used siRNA knockdown (KD) in MDA-MB-231 and MDA-MD-157 cells. The results of these studies indicate that CYP1B1 expression augments multiple phenotypes associated with aggressive characteristics of TNBC, and that elevated expression in breast tumors is associated with poor prognosis. Our findings that CYP1B1 KD can increase chemosensitivity supports the idea that therapeutic targeting of this enzyme, in combination with chemotherapeutic drugs, may provide a novel targeted approach to adjuvant or neoadjuvant therapy against certain forms of highly metastatic breast cancer.

## 2. Results

### 2.1. CYP1B1 Knockdown Decreases Mesenchymal Phenotypes in Triple-Negative Breast Cancer Cell Lines

**Figure 1:**
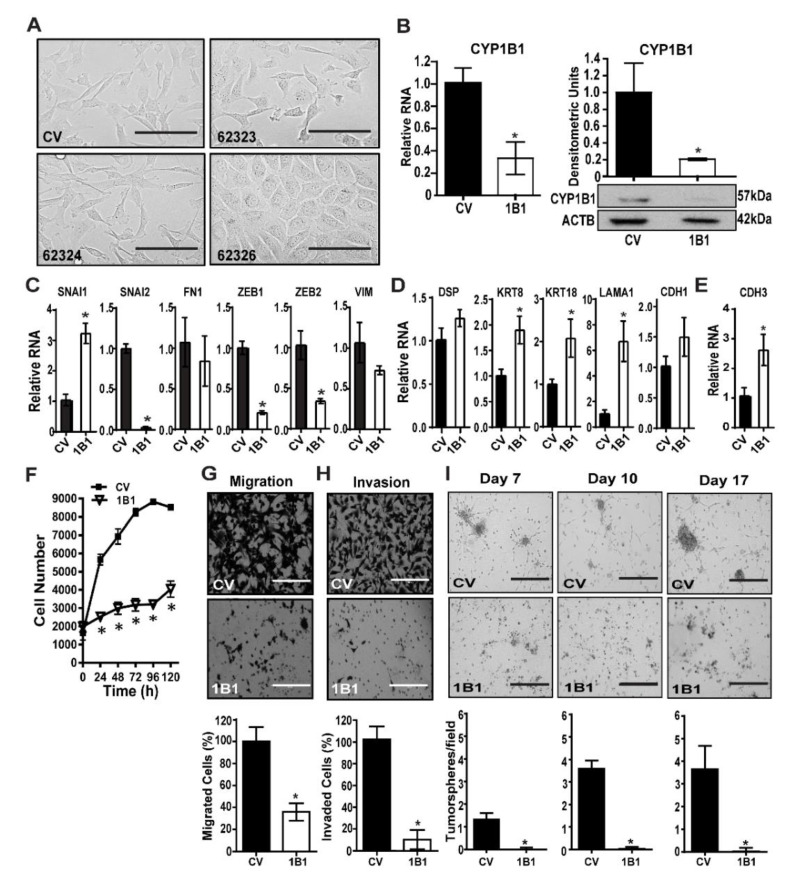
Stable CYP1B1 knockdown decreases mesenchymal phenotypes in MDA-MB-231 cells. (**A**) MDA-MB-231 cells were transduced with control vector (CV) or CYP1B1 short hairpin RNA (shRNA) Lentiviral Transduction Particles (62323, 62324, or 62326). Representative microscopic images at 200× magnification; scale bar, 200 µm. (**B**) Stable KD of CYP1B1 cell line 62326. Cells grown for more than 5 passages were analyzed for RNA (**left panel**) or protein normalized to ACTB (**right panel**). Values are the mean ± SD, n = 3, relative to CV, set at a value of 1.0. The * indicates a significant difference (*p* ≤ 0.05) between CV and CYP1B1 KD (shRNA) cells by Student’s *t*-test. (**C**) Quantitative PCR (qPCR) analysis of mesenchymal marker transcripts. Values are the mean ± SD, n = 3, relative to CV. The * indicates a significant difference (*p* ≤ 0.05) between CV and CYP1B1 KD cells (62326, here and below) by Student’s *t*-test. (**D**) qPCR analysis of epithelial marker transcripts. Values are the mean ± SD, n = 3, relative to CV. The * indicates a significant difference (*p* ≤ 0.05) between CV and CYP1B1 KD cells by Student’s *t*-test. (**E**) qPCR analysis of CDH3 transcripts. Values are the mean ± SD, n = 3, relative to CV. The * indicates a significant difference (*p* ≤ 0.05) between CV and CYP1B1 KD cells by Student’s *t*-test. (**F**) Cell proliferation was assayed at the indicated times. Values are the mean ± SD, n = 7; * indicates a significant difference (*p* ≤ 0.05) between CV and 1B1 KD cells by two-way analysis of variance (ANOVA) followed by Tukey’s multiple comparisons. (**G**) Cell migration. **Top**, microscopic images of invading cells; scale bar, 200 µm **Bottom**, results expressed as a percentage of CV; values are the mean ± SD, n = 5. The * indicates a significant difference (*p* ≤ 0.05) between CV and CYP1B1 KD cells by Student’s *t*-test. (**H**) Cell invasion through Matrigel. **Top**, microscopic images of invading cells scale bar, 200 µm. **Bottom,** results expressed as percentage of CV; values the mean ± SD, n = 5. The * indicates a significant difference (*p* ≤ 0.05) between CV and CYP1B1 KD cells by Student’s *t*-test. (**I**) Multiple spheroid formation. **Top**, microscopic images of CV and CYP1B1 KD cells grown in Matrigel suspension at the indicated days in culture; scale bar, 400 µm. **Bottom,** values are the mean ± SD, n = 3; * indicates a significant difference (*p* ≤ 0.05) between CV transduced cells and 1B1 shRNA transduced cells by Student’s *t*-test at each time.

MDA-MB-231 cells were transduced with control vector (CV) or one of three CYP1B1 shRNA-expressing lentiviruses. CYP1B1 shRNA (62323 or 62324) transduced cells displayed some cells with a rounded cell morphology. CYP1B1 shRNA (62326) transduced cells showed a marked change in cell morphology, growing as a monolayer with rounded shape and increased cell-to-cell contact (Figure 1A). An initial screen demonstrated that the CYP1B1 shRNA transduced cells decreased CYP1B1 protein by 48% (62323), 33% (62324) and 76% (62326) compared to the CV. Based on these results, the CV and 62326 cell lines were selected for further study. The stability of cell line 62326 was checked after multiple passages. Relative to CYP1B1 levels in CV cells, CYP1B1 RNA remained significantly decreased by 68% in 62326 cells (Figure 1B, left); protein remained significantly decreased by 80% (Figure 1B, right). Analysis of a select subset of mesenchymal regulators and markers showed aspects of mesenchymal-epithelial transition (MET), consistent with the changes in mesenchymal phenotypes. However, while certain regulators of the mesenchymal phenotypes, namely SNAI2, ZEB1 and ZEB2 were strongly decreased by CYP1B1 KD, SNAI1 was elevated 3-fold. Moreover, VIM and FN1 were not significantly decreased (Figure 1C). Parallel measures of epithelial cell markers showed similar results. KRT8, KRT18 and LAMA1 were each significantly increased, while CDH1 was not (Figure 1D). Levels of CDH3 were significantly elevated by nearly 3-fold (Figure 1E) suggesting that these CYP1B1 KD cells may be in an intermediate state [19], with transition between mesenchymal and epithelial characteristics, termed epithelial-mesenchymal plasticity [20,21]. The CYP1B1 KD cells showed a markedly slower rate of proliferation in comparison to CV transduced cells (Figure 1F). This phenotype was replicated by siRNA KD of CYP1B1 in both MDA-MB-231 and MDA-MB-157 cells. The siRNA transfection of these cells did not decrease viability at 24 h. (Supplementary Figure S1A–C). Furthermore, the siRNA KD cells had a much slower growth rate. Levels of caspase 3 were modestly (60%), yet significantly elevated. Caspase 3 cleavage was also increased about 67%. However, no cleavage of poly(ADP-ribose) polymerase (PARP) protein was detected (Supplementary Figure S1D). Thus, it does not appear that apoptosis accounts for all the observed differences in cell proliferation rates.

Consistent with the altered mesenchymal to epithelial morphology (Figure 1A and Figure S1B), CYP1B1 KD resulted in a robust and significant decrease in cell migration (Figure 1G and Figure S1E), and the effects to decrease invasion through Matrigel were even greater (Figure 1H and Figure S1F). Multiple spheroid formation in Matrigel suspension was much greater in CV cells than in CYP1B1 KD cells (Figure 1I), consistent with lowered growth rates and loss of invasive phenotypes. Collectively, these results indicate an important role of CYP1B1 in the highly invasive phenotypes of TNBC cells.

### 2.2. CYP1B1 Suppression Is Associated with Increased CLDN Expression in CL TNBC Cell Lines

**Figure 2:**
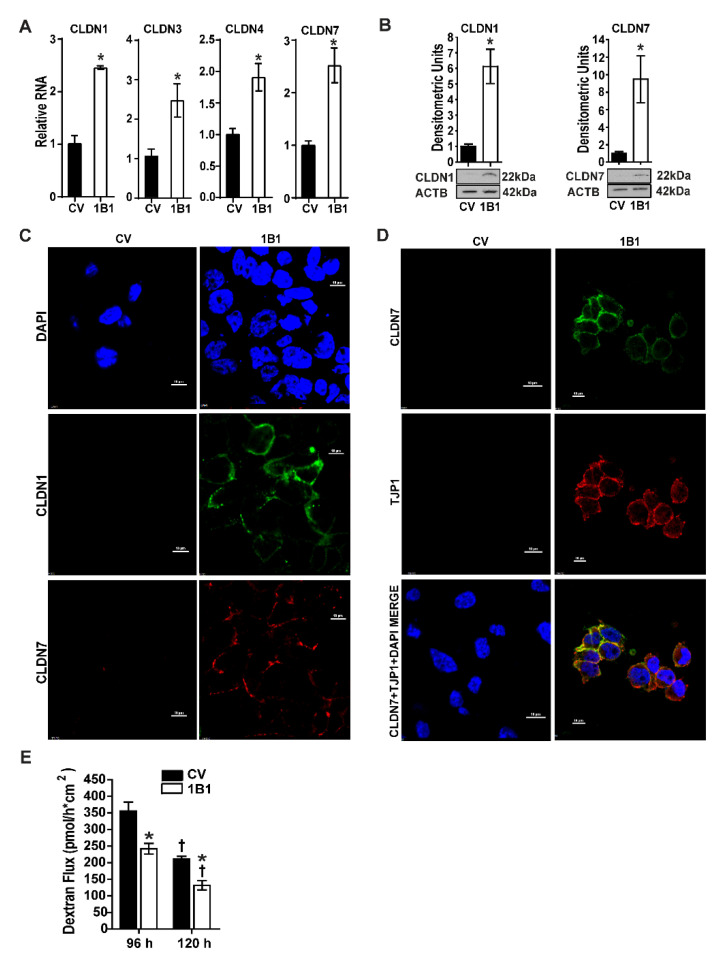
CYP1B1 suppression is associated with increased expression of claudins in CL triple-negative breast cancer (TNBC) cells. (**A**) qPCR analysis of CLDN1, CLDN3, CLDN4, and CLDN7 transcripts. Values are the mean ± SD, n = 3, relative to CV, set at a value of 1.0. The * indicates a significant difference (*p* ≤ 0.05) between CV and CYP1B1 KD cells by Student’s *t*-test. (**B**) Protein levels of CLDN1 and CLDN7. CLDN1 and CLDN7 densities were normalized to ACTB. Values are the mean ± SD, n = 3, relative to CV The * indicates a significant difference (*p* ≤ 0.05) between CV and CYP1B1 KD by Student’s *t*-test. (**C**) Confocal microscopic images of CV and CYP1B1 KD cells; CLDN1 (green) and CLDN7 (red) immunofluorescent staining. Nuclei were visualized with DAPI (blue), magnification 600×, scale bar, 10 µm. See methods for instrument parameters. (**D**) Confocal microscopic images of CV and CYP1B1 KD cells; CLDN7 (green) and TJP1 (red) immunofluorescent staining. Nuclei were visualized with DAPI (blue). Representative confocal microscopic images at 600× magnification, scale bar, 10 µm. (**E**) Paracellular permeability measured in CV or CYP1B1 KD cells cultured for 96 h or 120 h. Values are the mean ± SD, n = 3. The * indicates a significant difference between CV and CYP1B1 KD cells at the same time point, and the † indicates a significant between 96 h and 120 h of the same cell line; *p* ≤ 0.05 using two-way ANOVA followed by Tukey’s multiple comparisons.

As high CYP1B1 activity is associated with mesenchymal TNBC cells lines [16] having a CL phenotype [17], we explored the relationship between CYP1B1 suppression and CLDN expression. Levels of CLDN1 RNA and protein were strongly elevated in CYP1B1 KD cells (Figure 2A,B and Figure S2A–C). Moreover, the characteristic low expression of CLDN3, CLDN4 and CLDN7 RNA in CL cells was elevated in CYP1B1 KD MDA-MB-231 (Figure 2A and Figure S2A) and CYP1B1 KD MDA-MB-157 cells (Supplementary Figure S2B). Immunoblot of CLDN7 protein revealed a 9-fold increase in this protein in CYP1B1 KD MDA-MB-231 cells (Figure 2B and Figure S2D). Similar increases of CLDN1 and CLDN7 proteins were observed by indirect immunofluorescence in comparisons of CV and CYP1B1KD MDA-MB-231 cells. Representative confocal microscope images showed increased CLDN1 and CLDN7 immunofluorescence staining around the perimeter of the cells (Figure 2C and Figure S2C,D). These increases corresponded with the change of cell morphology from mesenchymal to epithelial (Supplementary Figure S2C,D). Both CLDN1 and CLDN7 proteins were organized at the apical-lateral borders at points of cell-to-cell contact in CYP1B1 suppressed cells in comparison to the CV cells (Figure 2C). Furthermore, dual detection of CLDN7 and tight junction protein 1 (TJP1), a scaffolding protein essential for proper tight junction formation [22], indicated that both proteins organized at the apical-lateral borders of the KD cells, especially at areas of cell-to-cell contact (Figure 2D). Co-localization of these proteins was determined by correlation analyses. The Pearson correlation coefficient was 0.71; the Mander’s overlap coefficients were c1 = 0.977 and c2 = 0.992. Both approaches were supportive of co-localization. To determine whether these observed increases provided the cells with improved tight junction barrier function, we measured paracellular permeability. At both 96 h and 120 h post plating, CYP1B1 KD cells showed significant decreases of paracellular flux in comparison to CV cells (Figure 2E). Together, these data revealed that CYP1B1 KD is associated with increased CLDN levels in CL-low TNBC cell lines, resulting in increased adhesion and tight junction function.

### 2.3. Association of RNA Levels of CYP1B1 and CLDN7 in Clinical Data of Breast Cancer Samples

**Figure 3:**
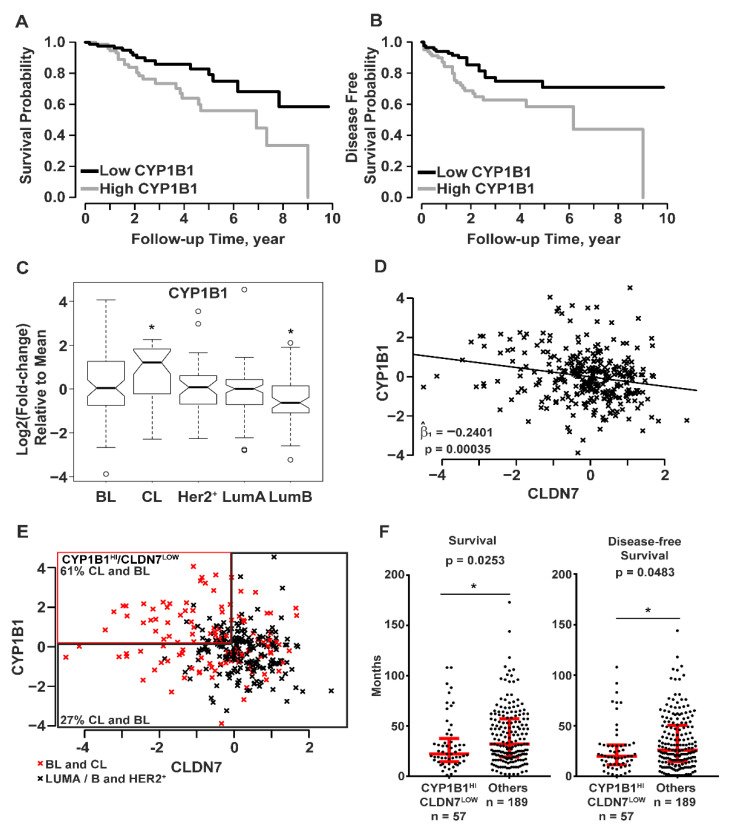
Patient tumor sample data (UNC337) indicate that CYP1B1 is associated with poor prognosis and reduced CLDN7 expression. (**A**–**F**) Data from breast cancer samples were grouped into the five intrinsic subtypes: basal-like (BL), claudin-low (CL), human epidermal growth factor receptor (HER2^+^), luminal A (LumA), and luminal B (LumB); UNC337 patient tumor sample database [5]. (**A**,**B**) Patient tumor sample data were separated into tertiles (104 high, 103 middle, and 103 low) of CYP1B1 RNA levels. (**A**) Survival probability was calculated using Kaplan-Meier analysis. Tumor patients with high CYP1B1 RNA levels had decreased probability of survival (Mantel-Cox test; *p* = 0.0115). (**B**) Disease-free survival was calculated using Kaplan-Meier analysis. Tumor patients with high CYP1B1 RNA levels have decreased probability of disease-free survival (Mantel-Cox test; *p* = 0.0111). (**C**,**D**) All 331 sample gene expression is shown as log2 (fold-change) relative to the mean expression of all samples. (**C**) Mean CYP1B1 RNA levels are elevated in CL tumor samples in comparison to all other samples. Boxplots show the inter-quartile range (IQR) within the box. The IQR is 25th percentile (Quartile 1) to 75th percentile (Quartile 3). The notch shows the 95% confidence interval of the median, and the whiskers include values within ±1.5 IQR and 99.3% of the data points. Two-sample *t*-test (*p* ≤ 0.05) of the means were corrected for multiple comparisons using Bonferroni’s correction. (**D**) CYP1B1 RNA levels predict CLDN7 RNA levels The coefficient of the linear regression line, Beta one hat ($\hat{\beta }$_1_), is the estimate of the slope of the line that best fits the data points. The significance of this value was determined by *t*-test. (**E**) Combined BL and CL tumor samples contain more CYP1B1^HI^/CLDN7^LOW^ samples than combined (LumA+ B+ HER2^+^) samples. Samples with >10% increase in CYP1B1 RNA levels and >10% decrease in CLDN7 RNA levels were classified as CYP1B1^HI^/CLDN7^LOW^ relative to RNA levels in all other samples. Sixty-one percent of CYP1B1^HI^/CLDN7^LOW^ samples were subtyped as CL or BL, while only 27% of all other samples were CL or BL. (**F**) Patients with CYP1B1^HI^/CLDN7^LOW^ have decreased overall survival and disease-free survival. Red lines and whiskers show the median ± IQR. The * indicates a significant difference (*p* ≤ 0.05) from comparison of CYP1B1^HI^/CLDN7^LOW^ to all other samples by the Mann-Whitney test.

Microarray gene expression data with corresponding clinical data of breast cancer samples were accessed from the UNC Microarray Database, UNC337 [5]. Breast cancer samples were divided into tertiles by CYP1B1 RNA levels: low, medium, and high. Samples with the highest CYP1B1 RNA levels were compared to samples with the lowest CYP1B1 RNA levels. Kaplan-Meier analysis demonstrated that breast cancer patients with high CYP1B1 expression had decreased probability of overall survival (Figure 3A) and disease-free survival (Figure 3B) than those with low CYP1B1 expression. Further analysis considered the expression of CYP1B1 within five intrinsic subtypes: BL, CL, HER2^+^, LumA, and LumB [5,8]. CYP1B1 expression was significantly higher in CL samples, and significantly lower in LumB samples (Figure 3C). Linear regression analysis identified a significant negative regression coefficient representing CYP1B1 and CLDN7 RNA levels (Figure 3D). Grouped by CYP1B1^HI^/CLDN7^LOW^, 61% of CL and BL samples contained more CYP1B1^HI^/CLDN7^LOW^ samples while 27% of all other samples were CYP1B1^HI^/CLDN7^LOW^ (Figure 3E). Comparison of CYP1B1^HI^/CLDN7^LOW^ CL and BL samples to all other samples revealed a significant decrease in the median survival and disease-free survival of patients identified as CYP1B1^HI^/CLDN7^LOW^ (Figure 3F). Overall, these analyses of this set of clinical data showed a negative correlation of expression of CYP1B1 and CLDN7 in breast cancer, consistent with our findings in CL TNBC (Figure 2). Moreover, these results indicate that patients with CYP1B1^HI^/CLDN7^LOW^ tumors may have a poorer prognosis.

### 2.4. CYP1B1 Suppression Enhances the Sensitivity of MDA-MB-231 Cells to Chemotherapeutic Drugs

**Figure 4:**
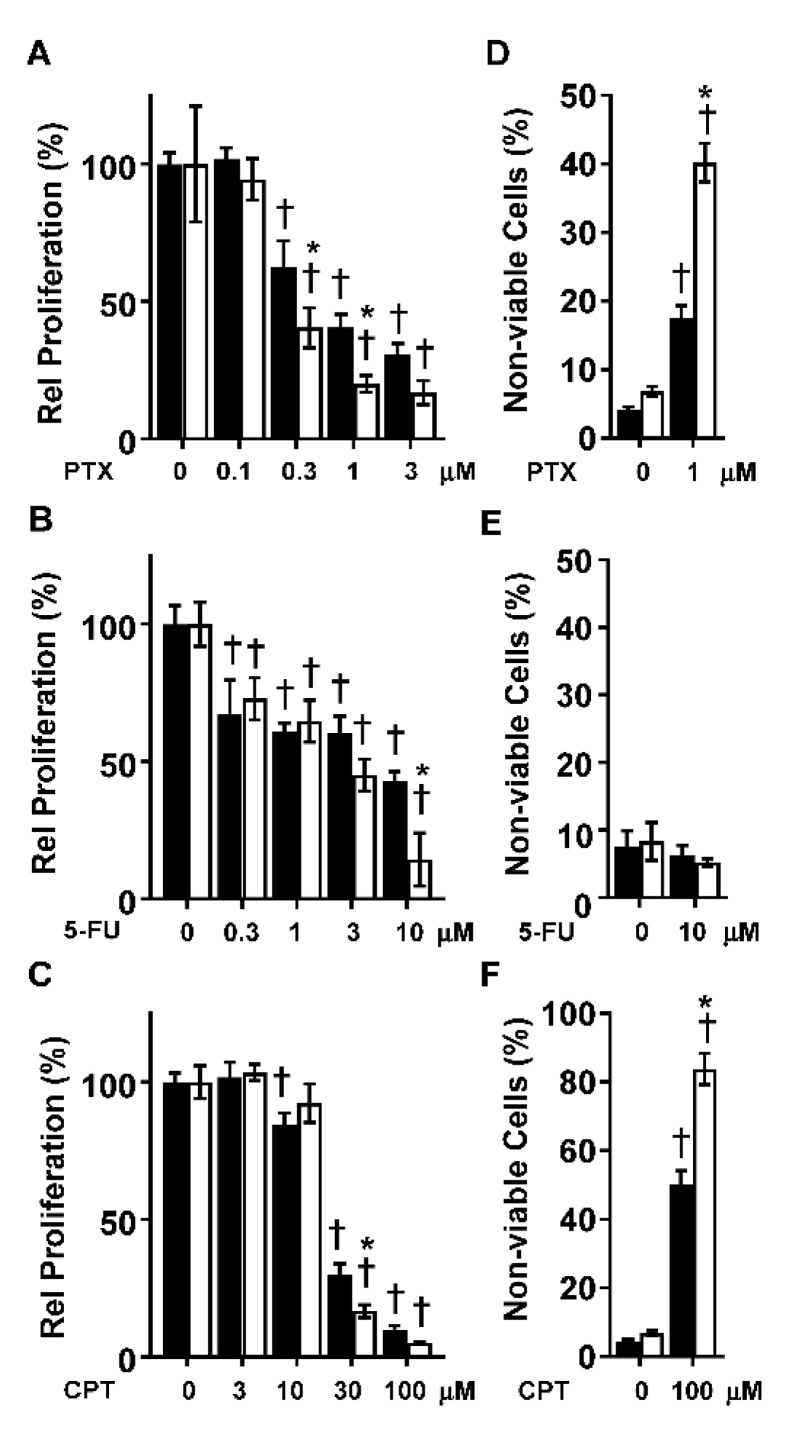
Suppression of CYP1B1 sensitizes MDA-MB-231 cells to the effects of paclitaxel (PTX), 5-fluorouracil (5-FU), and cisplatin (CPT). (**A**) CV (black bars) and CYP1B1 KD (white bars) cells were treated with increasing concentrations of PTX (72 h) before cell proliferation was determined. Values are the mean ± SD, n = 5. (**B**) CV and CYP1B1 KD cells were treated with increasing concentrations of 5-FU (72 h) before cell proliferation was determined. Values are presented as the mean ± SD, n = 5. (**C**) CV and CYP1B1 KD cells were treated with increasing concentrations of CPT (72 h) before cell proliferation was determined. Values are presented as the mean ± SD, n = 6. For (**A**–**C**), relative proliferation was calculated by setting the vehicle control for each cell line to 100%. The * indicates a significant difference (*p* ≤ 0.05) in comparison to CV within the same concentration of drug and the † indicates a significant difference from vehicle within the same cell line by two-way ANOVA followed by Tukey’s multiple comparisons. (**D**) CV and CYP1B1 KD cells were treated with vehicle or PTX (1 μM, 72 h) followed by measurement of dead cells. Values are the mean ± SD, n = 5. (**E**) CV and CYP1B1 KD cells were treated with control vehicle or 5-FU (10 μM, 72 h) followed by measurement of dead cells. Values are the mean ± SD, n = 3. (**F**) CV and CYP1B1 KD cell were treated with control vehicle or CPT (100 μM, 72 h) followed by measurement of dead cells. Values are the mean ± SD, n = 5–9. The * indicates a significant difference (*p* ≤ 0.05) in comparison to CV within the same concentration of drug and the † indicates a significant difference from vehicle within the same the cell line using two-way ANOVA followed by Tukey’s multiple comparisons.

To determine whether CYP1B1 KD altered the sensitivity of MDA-MB-231 cells to chemotherapeutic drugs, MDA-MB-231 CV or CYP1B1 KD cells were treated with paclitaxel (PTX), 5-fluorouracil (5-FU) or cisplatin (CPT). Treatment of MDA-MB-231 CV cells with PTX, 5-FU or CPT resulted in a concentration-dependent decrease in cell proliferation (Figure 4A–C). Because of the marked effect of CYP1B1 KD on cell proliferation (Figure 1F), a significant interaction of KD and chemotherapeutic agent was only observed at specific drug concentrations. Compared to the CV cells, proliferation was lower in the CYP1B1 KD cells following 0.3, 1 and 3 μM PTX treatment (Figure 4A and Figure S3A), 10 μM 5-FU (Figure 4B), and 30 μM CPT (Figure 4C). Because cell proliferation reflects both the rates of cell replication and cell death, and because CYP1B1 KD had such a large effect on the rate of cell replication (Figure 1F), we determined cell viability 72 h post treatment with a single effective concentration of each agent. In untreated cells, the percentage of non-viable cells was less than 8%, with no significant difference between CV and CYP1B1 KD cells (Figure 4D–F). Treatment of cells with 1 μM PTX significantly increased the percentage of non-viable cells from 13% in CV cells to 40% in CYP1B1 KD cells (Figure 4D). In contrast, treatment with 10 μM 5-FU resulted in no change in the percentage of non-viable cells in either CV or CYP1B1 KD cells (Figure 4E). Treatment of cells with 100 μM CPT significantly increased the percentage of non-viable cells from 50% in CV cells to 82% in CYP1B1 KD cells (Figure 4F). Preliminary studies of the highly potent and selective CYP1B1 inhibitor 2,3′,4,5′-tetramethoxystilbene (TMS) [23] revealed that TMS decreased cell invasion in a concentration-dependent manner. Moreover, co-treatment of TMS and 5-FU showed a significantly greater decrease in cell proliferation than did 5-FU alone (Supplementary Figure S4A,B).

## 3. Discussion

CYP1B1 KD in TNBC cells induced changes from mesenchymal to epithelial morphology. This was accompanied by decreased expression of known regulators of the mesenchymal phenotype and increased expression of epithelial markers. Of importance, robust changes in cell proliferation, migration, and invasion were observed. Multiple spheroid formation was markedly and significantly decreased in the CYP1B1 KD cells. proliferation reflects both the rates of cell replication and cell death, and because CYP1B1 KD had such a large effect on the rate of cell replication (Figure 1F), we determined cell viability 72 h post treatment with a single effective concentration of each agent. In untreated cells, the percentage of non-viable cells was less than 8%, with no significant difference between CV and CYP1B1 KD cells (Figure 4D–F). Treatment of cells with 1 μM PTX significantly increased the percentage of non-viable cells from 13% in CV cells to 40% in CYP1B1 KD cells (Figure 4D). In contrast, treatment with 10 μM 5-FU resulted in no change in the percentage non-viable cells in either CV or CYP1B1 KD cells (Figure 4E). Treatment of cells with 100 μM CPT significantly increased the percentage of non-viable cells from 50% in CV cells to 82% in CYP1B1 KD cells (Figure 4F). Preliminary studies of the highly potent and selective CYP1B1 inhibitor 2,3′,4,5′-tetramethoxystilbene (TMS) [23] revealed that TMS decreased cell invasion in a concentration-dependent manner. Moreover, co-treatment of TMS and 5-FU showed a significantly greater decrease in cell proliferation that did 5-FU alone (Supplementary Figure S4A,B). Collectively, these results indicate that CYP1B1 KD results in MET, characterized as a population of cells in which the migratory and spindle-shaped mesenchymal cells transition to a more organized and polarized epithelial cell morphology, with less aggressive characteristics [24].

In a study of inflammatory breast cancer (IBC) clinical data, CYP1B1 and AHR RNA were positively correlated and elevated in IBC samples. Expression levels of each positively correlated with expression of WNT5A/B and β-catenin [25], known downstream effectors of mammary tumors induced by AHR ligand activation [26]. Expression of WNT5A/B and β-catenin were correlated with markers of the cancer stem cell (CSC) phenotype, and AHR-antagonist treatment of MDA-MB-231 or SUM149 breast cancer cells resulted in significantly lower levels of WNT5A RNA. Sherr and colleagues [25,27,28,29] developed a model of TNBC where AHR activation drives properties associated with tumor progression including migration, invasion, expression of CSC markers, and CSC characteristics of enhanced spheroid formation, tumor formation in NOD/SCID mice, and chemoresistance. In this model, the AHR increases expression of indoleamine 2,3 dioxygenase (IDO)/tryptophan dioxygenase (TDO) enzymes to establish an IDO/TDO-AHR regulatory loop [27]. In this feed-forward signaling loop, IDO/TDO-catalyzed metabolism of tryptophan produces kynurenine, an AHR ligand, which acts to increase tumor aggression. This loop is autoregulated by AHR-dependent elevation of the AHR repressor, and CYP1A1 and CYP1B1 enzymes that metabolize kynurenine ligands to decrease their bioavailability [29], thus forming a cancer progression circuit where AHR-regulated CYP1B1 levels correlate with AHR activity. In this model, expression of CYP1B1 is viewed as a surrogate or biomarker of AHR signaling and hence the activity of this cancer progression circuit [25,29].

This model predicts that increases of CYP1B1 would increase AHR ligand metabolism and decrease AHR activity and aggressive phenotypes, while decreases in CYP1B1 would increase ligand availability, AHR activity, and aggressive phenotypes. Two studies contradict this model and indicate that CYP1B1 expression may play a significant role in tumor progression. In a study by Kwon et al. [30], ectopic expression of CYP1B1 in either ER- MCF-10F or ER+ MCF7 cell lines drove increased expression and nuclear localization of β-catenin, mesenchymal morphology, EMT-associated gene expression, and invasion. MCF-10F cells, while immortal, are neither transformed nor tumorigenic. Thus, while the establishment of the IDO/TDO tumor progression circuit seems unlikely, the overexpression of CYP1B1 nonetheless drove EMT in these cells [30]. Moreover, overexpression of CYP1B1 is not known to activate the AHR, and treatment of breast cancer cell lines with 4-hydroxyestradiol, the primary estrogen product of CYP1B1, results in effects similar to ectopic CYP1B1 expression [30]. Plausible targets of estrogen metabolites include ERβ and G protein estrogen receptor (GPER) [31]. Interestingly, GPER is reported to regulate CYP1B1 expression in ER- breast cancer and modulation of both GPER and CYP1B1 alter tumor cell growth in vitro and in vivo [32]. Thus, regulation of CYP1B1 by pathways in addition to the AHR may be important determinants to its expression and roles in tumor progression. The second line of evidence indicating that CYP1B1 contributes to these aggressive phenotypes is provided by the results of this current study where depletion of CYP1B1 blocked migration, invasion, and EMT. Collectively, these results indicate that CYP1B1 deserves additional study as an independent driver of aggressive tumor characteristics in TNBC.

Loss of tight junction proteins and lack of cell-to-cell contact are characteristics of multiple carcinomas, including TNBC. These features are viewed as essential for cellular invasion and migration that is necessary for metastasis [33]. CLDN proteins create tight junctions between cells, regulating paracellular permeability. Because MDA-MB-231 and MDA-MB-157 are characterized as claudin low TNBC cell lines and CYP1B1 KD cells displayed epithelial-like cell morphology and high frequency of cell-to-cell contacts, we measured the expression of CLDN RNAs and proteins. In CYP1B1KD cells, RNA levels of CLDN1, CLDN3, CLDN4 and CLDN 7 were elevated, as were proteins for CLDN1 and CLDN7. Confocal microscopy revealed organized cells with both CLDN1 and TJP1 expressed at the apical lateral borders. Moreover, functional tight junctions were demonstrated by decreases of paracellular permeability. Collectively, these data indicate that CYP1B1 contributes to the CL phenotype observed in the same TNBC cell lines.

To explore the possible clinical significance of CYP1B1 expression in breast cancer and CL breast cancer, we analyzed the UNC337 Microarray Database [5]. Analysis of the 311 tumor samples showed significant differences in overall and disease-free survival when the samples were grouped by tertiles into either high versus low expression of CYP1B1. Analysis of CYP1B1 expression by previously assigned intrinsic breast cancer subtypes [5] showed significantly higher expression in the CL subtype and significantly lower expression in the luminal B subtype. Analysis of all tumor samples showed a significant negative regression coefficient for CYP1B1 and CLDN7 expression values, consistent with our cell-based experiments. Sorting all samples by CYP1B1^HI^/CLDN7^LOW^ transcript levels revealed that 61% of the samples meeting this criterion were independently classified as either CL or BL. This finding is consistent with recent studies investigating CL as a complex phenotype rather than a novel intrinsic breast cancer subtype [13,14]. Overall, the CL breast tumors appear to reflect the characteristics of their cell of origin [14], and stratification of CL status does not indicate a poorer survival within an intrinsic subtype [13]. Fougner and colleagues [13] report a much greater proportion of CL tumors in the BL (14.6%), than in the HER2^+^ (0.9%), LumA (1.3%) or LumB (0.6%) intrinsic subtypes.

Despite such controversies related to CLDN expression and function in tumorigenesis, several studies indicate that increased CLDN1, CLDN4, or CLDN7 can act as a suppressor of metastasis, and a potential inducer of MET in breast, lung, colon, pancreatic, gastric epithelial and nasopharyngeal carcinoma cells [24,34,35,36,37]. Similarly, CLDN16 overexpression in MDA-MB-231 cells results in an epithelial-like cell shape, increased frequency of cell-to-cell contact, and attenuation of aggressive phenotypes [38]. Conversely, reduced expression of CLDN1 and CLDN7 correlates with invasion and metastases in gastric epithelial cells and carcinoma of the esophagus, respectively [39,40]. In the current study, both overall and disease-free survival were significantly lower in the samples classified as CYP1B1^HI^/CLDN7^LOW^. Thus, this simple two-factor classification (CYP1B1^HI^/CLDN7^LOW^) may be sufficient to identify breast cancer patients with poorer prognostic outcomes and aid in the development of novel targeted treatments for these aggressive cancers.

TNBC has a poor prognosis, in part, relating to its aggressive phenotypes and resistance to chemotherapy and radiation [41]. Here we showed that suppression of CYP1B1 results in dose-dependent decreases in the rate of proliferation of MDA-MB-231 cells to multiple chemotherapeutic agents including PTX, 5-FU and CPT relative to control cells. Further, these CYP1B1-drug interactions displayed differing effects on cell viability. For PTX and CPT, CYP1B1 KD enhanced chemical-dependent cytotoxicity, while for the antimetabolite 5-FU, CYP1B1 KD interacted to affect cell proliferation, but not cell viability. Thus, CYP1B1 modulation may affect multiple mechanisms that provide chemotherapeutic benefit in breast cancer treatment. Chemical inhibitors of CYP1B1 are reported to increase the sensitivity of TNBC cell lines to CPT [42] and other compounds in several tumor types [43]. Such inhibition is also reported to impart cardioprotective effects [43] indicating that there may be added benefits to chemotherapy approaches that include CYP1B1 inhibitors.

The mechanisms of chemoresistance are complex [44,45]. In some cases, CYP1B1 may act to alter chemotherapeutic disposition and thereby drug efficacy [46]. However, the data presented here and by others [25,30,32] support the idea that CYP1B1 directly contributes to complex cancer phenotypes including chemoresistance that may not directly involve drug metabolism. For example, the potent and selective CYP1B1 inhibitor, TMS, decreases cell growth and migration, as well as tumor growth in xenograft models in vivo [30,32,47]. Consistent with these reports, our studies of TMS revealed dose-dependent decreases in cell invasion and cell proliferation. Cells co-treated with 5-FU and TMS showed further decreases in cell proliferation. Overall, elevated expression of CYP1B1 in TNBC cells appears to enhance mesenchymal and claudin-low characteristics while knockdown or inhibition of this enzyme weakens their associated invasive and chemoresistant properties. These observations point towards novel therapeutic strategies for certain subtypes of breast cancer.

The results of the current study, while compelling, are limited in that they rely on the manipulation of CYP1B1 levels in a subset of breast cancer cell lines. These findings should be strengthened and expanded by future studies using patient derived cancer cells, as well as in vivo experimental studies of tumor growth and metastases, and patient-derived xenograft models. Such studies are necessary for a thorough preclinical evaluation of the importance of CYP1B1 to the aggressive phenotypes associated with TNBC.

## 4. Materials and Methods

### 4.1. Cell Culture

The MDA-MB-231 and MDA-MB-157 human triple-negative breast cancer cell lines (HTB-26 and HTB-24, ATCC, Manassas, VA, USA). were grown in Dulbecco’s Modified Eagle Medium (DMEM; 11960-044, Thermo Fisher Scientific, Waltham, MA, USA), supplemented with 2 mM glutamine (MIR6240, Mirus, Madison, WI, USA), 10% fetal bovine serum (FBS; S11550, Atlanta Biologicals, Nashville, TN, USA), 10,000 IU/mL penicillin and 10,000 µg/mL streptomycin (25300-054, Thermo Fisher Scientific) at 37 °C, 5% CO_2_ in a humidified incubator. Cell lines were previously tested for mycoplasma contamination. Experiments were performed in phenol red-free DMEM (31053-028, Thermo Fisher Scientific) supplemented with 2 mM glutamine and 10% FBS, except as noted.

### 4.2. Chemicals

PTX (T7402, Millipore Sigma, Burlington, MA, USA), 5-FU (F6627, Millipore Sigma), CPT (22-515-0, Thermo Fisher Scientific) and TMS (10038, Cayman Chemicals, Ann Arbor, Michigan, USA) were dissolved in dimethyl sulfoxide (DMSO; D2650, Millipore Sigma). The final concentration of DMSO in the media did not exceed 0.1%.

### 4.3. siRNA Transfection

For transient suppression of CYP1B1, MDA-MB-231 and MDA-MB-157 cells were seeded at 10,500 cells/cm^2^ in 12-well plates and incubated overnight. Following the manufacturer’s protocol, cells were transfected with CYP1B1 siRNA (s49828, Thermo Fisher Scientific) or non-specific siRNA using Oligofectamine (12252-011, Thermo Fisher Scientific). In brief, 20 μM siRNA stock solution (2.5 μL) was diluted with Opti-MEM (87.5 μL) (31985070, Thermo Fisher Scientific). Oligofectamine (3 μL) was diluted with Opti-MEM (7 μL). Dilutions were incubated for 10 min at room temperature, combined and mixed gently, then further incubated for 20 min at room temperature to allow complex formation. Opti-MEM (400 μL) was added to each well and then Oligofectamine/Opti-MEM mixture (100 μL) alone (mock) or containing the siRNA was added to the wells drop-wise. Following 4 h of incubation at 37 °C, 250 μL of recovery medium (phenol-red free medium, 30% FBS, 2 mM glutamine) was added to each well. Two siRNAs targeting CYP1B1, LT-1B1 (s49828, Thermo Fisher Scientific) and Q-1B1 (SI00002079, Qiagen, Germantown, MD, USA), were investigated for suppression efficiency. The Q-1B1 siRNA (100 nM) produced the highest suppression efficiency and was used for future experiments. The sequence for the sense strand Q-1B1 siRNA are as follows: sense strand 5′-GCAUGAUGCGCAACUUCUUTT-3′ and antisense strand 5′-AAGAAGUUGCGCAUCAUGCTG-3′.

### 4.4. CYP1B1 shRNA Transduction Cell Lines

MDA-MB-231 cells were transduced with five different lentiviral constructs containing shRNA specific for CYP1B1. To obtain stable CYP1B1 suppressed cells, MDA-MB-231 cells were seeded in a 12-well plate at a density of 4 × 10^4^ cells/well and incubated overnight. Medium was then replaced with medium containing 5 µg/mL polybrene and the cells were transduced with a multiplicity of infection of 2 transducing units. 48 h post-transduction, medium was replaced with medium containing 2 μg/mL puromycin. The following CYP1B1 MISSION shRNA Lentiviral Transduction Particles constructs (Sigma Millipore) were tested: TRC numbers are 62323, 62324, 62325, 62326, and 62327, or empty vector backbone (CV, SHC001V).

### 4.5. Quantitative PCR (qPCR)

Total RNA was isolated from cells using STAT-60 (CS502, Tel-Test, Friendswood, TX, USA). qPCR was performed as described [48] using the following conditions: denaturation at 95 °C for 15 min, followed by denaturation at 95 °C for 30 s with 40 cycles, annealing from 52–58 °C for 30 s (dependent on primer pair) and extension at 72 °C for 30 s. Gene expression was normalized to ACTB expression. Primer sets used in this study are provided in Supplementary Table S1.

### 4.6. Cell Proliferation Assays

Cell proliferation was assessed using the Fluoreporter Blue Fluorometric dsDNA quantitation method (F2962, Thermo Fisher Scientific) or MTT assay (CT01, Sigma Millipore). For the Fluoreporter assay, cell number was measured by using the Hoescht 33258 according to the manufacturer’s procedure. Fluorescence was measured at excitation of 360 nm and emission of 460 nm using a Synergy H1 Microplate Reader and Gen 5 software version 2.09 (BioTek, Winooski, VT, USA). For the MTT assay, the manufacturer’s instructions were followed.

### 4.7. Invasion and Migration Assay

The invasion and migration assays followed [49] with minor modifications. The invasion assay was performed using 24-well polyethylene terephthalate membrane transwell inserts with 8.0 μm pores (353493, Corning, Corning, NY, USA). Inserts were coated with Matrigel (1 mg/mL; 356237, Corning) and allowed to polymerize overnight at 37 °C. Matrigel was rehydrated for 2 h before addition of cells to the insert. Cells were seeded at 1 × 10^5^ cells/cm^2^ in phenol-red free, serum free DMEM in the upper chamber of the Matrigel coated insert. FBS (10%) was added to the lower chamber, basal side of the membrane. Inserts were incubated at 37 °C and cells were allowed to invade for 22 h. The non-invading cells on the apical side of the membrane were removed with a cotton swab. Cells that invaded through the Matrigel and attached to the basal side of the membrane were fixed in 100% methanol and stained with 0.05% crystal violet for 10 min and rinsed two times in distilled water. The inserts were visualized using an EVOS digital inverted microscope (Thermo Fisher Scientific) with 200× magnification. Images of 16 fields per insert were taken. Experiments were performed with five replicate inserts. The invaded cells on the basal side of the insert were counted manually and shown as the mean of 80 fields per treatment. The cell migration assay was performed identical to the invasion assay, excluding the use of Matrigel coating of transwell inserts.

### 4.8. Protein Extraction and Immunoblot

Whole cells were harvested and lysed in RIPA Buffer (0.1% SDS, 0.5% Na-deoxycholate, 50 mM Tris pH 8.0, 150 mM NaCl, 1% NP40, 5 mM EDTA pH 7.4) supplemented with 0.2 M PMSF and protease inhibitor cocktail (P8340, Millipore Sigma). Lysates were placed, processed and protein concentration determined as described [48]. The blots were incubated with antibodies to CYP1B1 (1:5000; rabbit; [50], CLDN1 (1:500; rabbit, 51-9000, Thermo Fisher Scientific) or CLDN7 (1:500; mouse, 37-4800, Thermo Fisher Scientific). ACTB antibody (1:40,000; mouse, A5441, Millipore Sigma) was used to control for loading differences. Blots were incubated with primary antibodies for 1 h at room temperature (CYP1B1 and ACTB antibodies) or overnight at 4 °C (CLDN1 and CLDN7 antibodies) with gentle agitation. Blots were washed for three 10 min periods with 1× TBS-T Buffer (0.1% Tween 20) and incubated with horseradish peroxidase conjugated secondary IgG antibodies (1:10,000; goat anti-rabbit, 111-035-003 or 1:10,000; goat anti-mouse, 115-035-003, Jackson ImmunoResearch, West Grove, PA, USA) for 1 h at room temperature with gentle agitation. Blots were washed for three 10 min periods with 1× TBS-T Buffer (0.1% Tween 20) and bands were visualized with the use of Clarity Western ECL (170-5061, Bio-Rad). NIH ImageJ software or Image Lab (Version 5.2., BioRad, Hercules, CA, USA) were used to quantitate the immunoblot images, normalizing the densitometric values to ACTB.

### 4.9. Morphology Assessment

Cells grown in culture were visualized using an EVOS Digital Inverted Microscope with 200× magnification for morphology assessment. Images of 10 fields per well were taken at random and counted for mesenchymal or epithelial morphology.

### 4.10. Trypan Blue Exclusion Assay

Cells collected by trypsinization were added to media containing 10% FBS. After centrifugation, the pellets were resuspended in 0.4% trypan blue (T8154, Sigma): PBS (1:1) immediately before four quadrants of cells were counted manually using a hemocytometer under an inverted phase contrast Zeiss Axiovert 100 microscope at 10× magnification. Non-viable cells (blue cells) and the total number of cells (live and dead) were counted in each quadrant before percent non-viable cells was calculated.

### 4.11. Indirect Immunofluorescence

Transduced cells were plated on Matrigel-coated (1 mg/mL; 356237, Corning) coverslips and incubated to 3 days. Cells were fixed in 4% formaldehyde (1008A, Tousimis, Rockville, MD, USA) for 5 min and permeabilized with 0.1% NP-40 (492018, Millipore Sigma) for 5 min. Coverslips were incubated for 30 min at room temperature with antibodies to CLDN1 (rabbit, 1:100; 71-7800, Thermo Fisher Scientific), CLDN7 (mouse, 1:100; 37-4800, Thermo Fisher Scientific) or TJP1 (ZO-1, mouse, 1:100; 33-9100, Thermo Fisher Scientific). Coverslips were washed for three 5 min periods with PBS and then incubated for 30 min at room temperature in a secondary antibody (1:50; donkey anti-rabbit Alexa Fluor 594, A-21207, Thermo Fisher Scientific or 1:50; donkey anti-mouse Alexa Fluor 488, 715-545-151, Jackson ImmunoResearch). Coverslips were mounted using Prolong Gold Anti-Fade with DAPI (P36935, Thermo Fisher Scientific) mounting solution and cured overnight at room temperature. Images were acquired using a Nikon A1 Laser-Scanning Confocal Microscope (Nikon, Melville, NY, USA), with a mercury lamp light source, standard detector filter cubes, 405 nm (DAPI), 488 nm (FITC), and 586 (TRITC) lasers, and a Plan Apo VC 60×/1.4 oil objective. All images were captured at a pixel dwell of 3.2 ms and size of 2048 × 2048 pixels. For comparison of CV and CYP1B1 KD for CLDN1 and CLDN7, all settings were the same: pixel size, 0.048 µM/pixel; Power amplification, Ch1 (DAPI) 10.5%, Ch2 (FITC) 9.0%, Ch3 (TRITC) 6.0%; pinhole size, 1.9 AU. All other settings were constant. For colocalization of CLDN7 and TJP1, the settings for CV capture where the same as above, except the pixel size was 0.060 µM/pixel. For images of the CYP1B1 samples, the image parameters were optimized for each fluorophore and antibody for co-localization analysis; power amplification, Ch1 (DAPI) 13.2%, Ch2 (FITC) 4.1%, Ch3 (TRITC) 6.0%; pinhole size, 1.2 AU. Co-localization was determined using the Pearson correlation coefficient and the Mander’s overlap coefficient (k1 = 0.74 and k2 = 0.72).

### 4.12. Multiple Spheroid Formation Assay

Cells (5000) were seeded in triplicate on Matrigel-coated (1 mg/mL; 356237, Corning) chamber slides in serum free medium, 2% suspension of Matrigel as described [51], and grown for up to 25 days. Images of 10 individual fields were captured for each of three chambers with the EVOS Digital Inverted Microscope at 100× magnification. Colonies (>50 cells) were counted manually.

### 4.13. Paracellular Permeability Assay

The paracellular permeability assay procedure was performed according to the previously described method [22]. Cells (1 × 10^5^) were plated in transwell inserts (353493, Corning) and grown to confluence. P buffer (10 mM HEPES, pH 7.4, 10 mM glucose, 1 mM sodium pyruvate, 145 mM NaCl, 3 mM CaCl_2_) was added to the basal chamber (600 μL) and to the apical chamber (164 μL). P buffer (60 μL) containing 40 kDa FITC-dextran (1 mg/mL) was added to the apical chamber and incubated at 37 °C for 3 h. Diffusion of the 40 kDa FITC-dextran from the apical chamber to the basal chamber was measured using the Synergy H1 Microplate Reader (excitation: 494 nm; emission: 521 nm).

### 4.14. Statistical Analysis of UNC337 Patient Tumor Sample Data

For all analyses, the normal/normal-like samples were removed from the dataset, leaving 311 samples: CL (n = 37), BL (n = 73), HER2^+^ (n = 39), LumA (n = 100), and LumB (n = 62), GSE 18229 [5]. These 311 samples were separated into tertiles by CYP1B1 RNA levels (low n = 103, medium n = 104, and high n = 104). Log-rank analyses of the CYP1B1-dependent differences in survival and disease-free survival were assessed using the Mantel-Cox test of the Kaplan-Meier curves generated in survival: Survival Analysis package in R * [https://cran.r-project.org] accessed on 5 February 2019. For each subtype, median log_2_ expression values were plotted as box and whisker plots relative to overall mean CYP1B1 expression level. Pairwise comparisons of the group mean values were performed using a *t*-test with Bonferroni’s correction for multiple testing. For analyses of CYP1B1 and CLDN7 expression, data are reported as a log_2_ (fold-change) relative to the mean expression value for all samples. Linear regression analysis was performed in R using CYP1B1 and CLDN7 data from all 311 samples. The relationship between levels of CYP1B1 and CLDN7 was estimated from the regression coefficient and tested by *t*-test. The CYP1B1^HI^/CLDN7^LOW^ population was defined as samples with >10% increase in CYP1B1 expression relative to the mean for all samples, and >10% decrease in CLDN7 expression relative to the mean for all samples. Significance of differences in median survival and disease-free survival between CYP1B1^HI^/CLDN7^LOW^ samples and all other samples was assessed using a non-parametric Mann-Whitney test due to non-normal distribution of survival data values.

### 4.15. Statistical Analysis

All experiments were performed with cells at different passage numbers and repeated at least two times. Major findings were replicated using two techniques for CYP1B1 KD (shRNA and siRNA) and certain results were replicated in multiple cell lines. Results reported for analyses of a single clinical breast data set are supported by functional studies performed in multiple cell lines. Statistical analysis was performed using GraphPad Prism software (version 7.03; GraphPad Software, La Jolla, CA, USA) and R * [https://cran.r-project.org] accessed on 5 February 2019. A two-tailed Student’s *t*-test was used to compare results from the control vector samples to the CYP1B1 shRNA transduced samples. Where appropriate, two-way analysis of variance (ANOVA) followed by Tukey’s multiple comparison tests were used to compare the effects of CYP1B1 expression levels on treatments. One-way ANOVA was used to compare results from the mock, non-specific, and CYP1B1 siRNA samples. Data were considered statistically significant with a *p* value of ≤ 0.05. Details of the methods used for clinical sample analysis are provided in that specific section and the figure legend.

## 5. Conclusions

In our studies of claudin-low TNBC cell lines, CYP1B1 appears to augment mesenchymal and CL phenotypes. Furthermore, knockdown of CYP1B1 lessens invasion, spheroid formation, and chemoresistance. These results indicate a need to better understand the function of CYP1B1 in the context of TNBC and the significance of the CYP1B1^HI^/CLDN7^LOW^ classification of breast tumors. Collectively, the present study has implications for the potential combined use of a CYP1B1 inhibitor with chemotherapy for both adjuvant and neoadjuvant treatment approaches to TNBC.

## Data Availability

The human tumor data were accessed from GSE 18229, kindly made available by Prat and colleagues [5]. The CYP1B1 antibody [50] is available from the corresponding author.

## Supplementary Materials

The following supporting information can be downloaded at: https://www.mdpi.com/article/10.3390/ijms23179670/s1.
