# Supplementary material for: CYP1B1 Augments the Mesenchymal, Claudin-Low, and Chemoresistant Phenotypes of Triple-Negative Breast Cancer Cells
Source: Int J Mol Sci. 2022 Aug 26;23(17):9670. doi: 10.3390/ijms23179670 (PMC9456208; doi:10.3390/ijms23179670)
Supplement: Supplementary file 1 [file ijms-23-09670-s001.zip › ijms-1877082-supplementary.pdf]

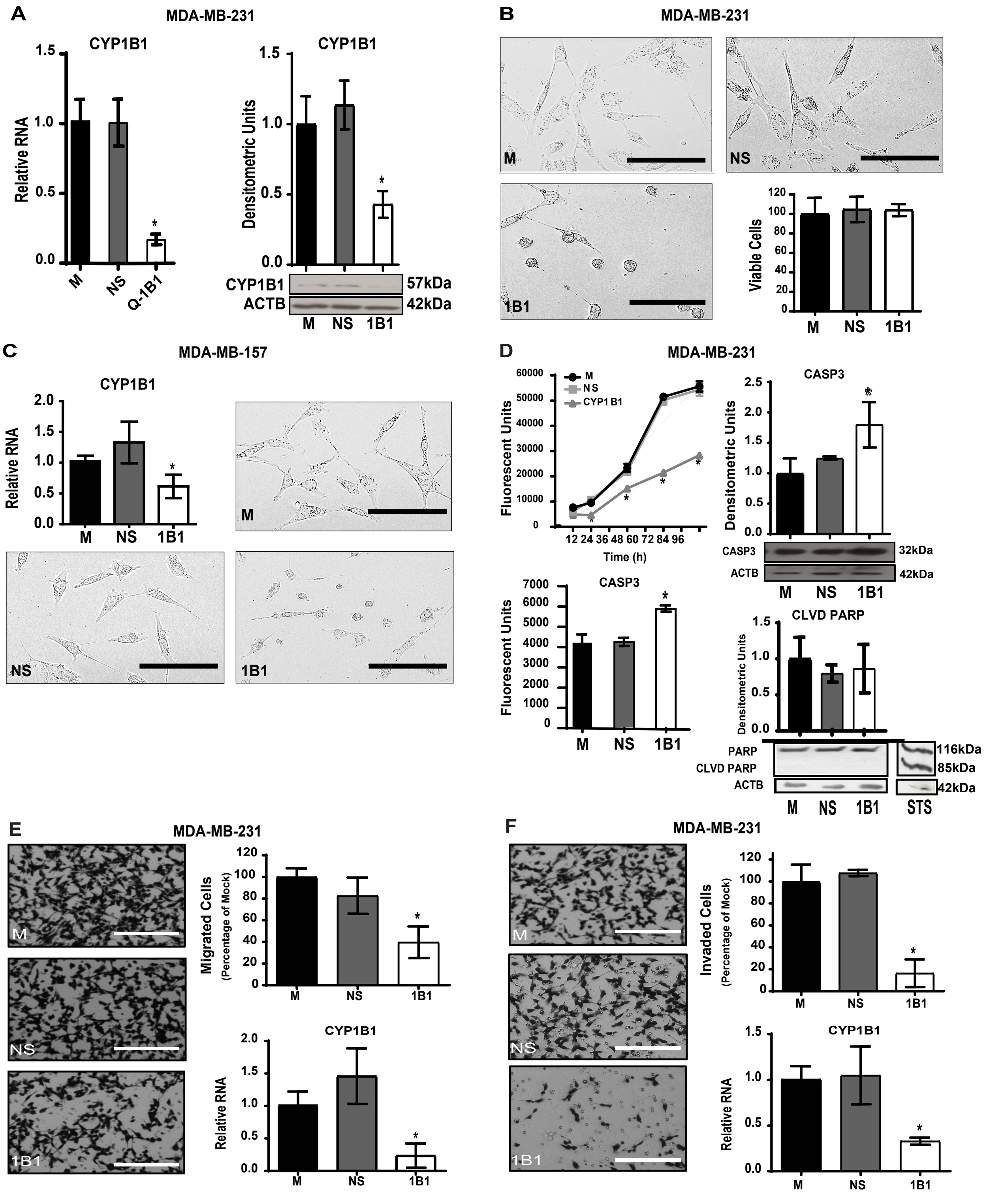
A
MDA-MB-231
CYP1B1
Relative RNA
M
NS
Q-1B1
CYP1B1
Densitometric Units
CYP1B1
57kDa
ACTB
42kDa
M NS 1B1
B
MDA-MB-231
M
NS
1B1
Viable Cells
M NS 1B1
C
MDA-MB-157
CYP1B1
Relative RNA
M NS 1B1
M
NS
1B1
D
MDA-MB-231
M
NS
CYP1 B1
Fluorescent Units
Time (h)
CASP3
Densitometric Units
CASP3
32kDa
ACTB
42kDa
M NS 1B1
CASP3
Fluorescent Units
M NS 1B1
CLVD PARP
Densitometric Units
PARP
CLVD PARP
ACTB
116kDa
85kDa
42kDa
M NS 1B1 STS
E
MDA-MB-231
M
NS
1B1
Migrated Cells
(Percentage of Mock)
M NS 1B1
CYP1B1
Relative RNA
M NS 1B1
F
MDA-MB-231
M
NS
1B1
Invaded Cells
(Percentage of Mock)
M NS 1B1
CYP1B1
Relative RNA
M NS 1B1

Supplementary Figure S1. CYP1B1 suppression is associated with loss of mesenchymal morphology and decreased rates of cell proliferation, invasion, and migration in triple negative breast cancer (TNBC) cell lines. MDA-MB-231 cells were transfected with CYP1B1 small interfering (siRNA) Qiagen (Q-1B1), non-specific siRNA (NS), or mock control (M); MDA-MB-157 cells were transfected with NS, M, or Q-1B1. **A, left panel,** qPCR analysis of CYP1B1 RNA. Values are the mean ± SD, n = 3, relative to M, set at a value of 1.0. The * indicates a significant difference (p ≤ 0.05) from NS cells by one-way ANOVA followed by Tukey's multiple comparisons. CYP1B1 siRNA (Q-1B1, white bar) was selected for further evaluation. **A, right panel,** CYP1B1 protein levels. Densitometric units of CYP1B1 were normalized to ACTB. The * indicates a significant difference (p ≤ 0.05) from NS cells by one-way ANOVA followed by Tukey's multiple comparisons. **B,** Cell morphology is shown in representative microscopic images at 200x magnification; scale bar, 200 μm. **B, lower right panel,** Cell viability was assessed using a trypan blue exclusion assay. Viable cells were expressed as a percentage of M. Data were analyzed by one-way ANOVA followed by Tukey's multiple comparisons. **C,** MDA-MB-157 cells were transfected with M, NS or Q-1B1 siRNAs. **C, upper left panel**, qPCR analysis of CYP1B1 RNA in MDA-MB-157 cells. Values are presented as the mean ± SD, n = 3, relative to M, set at a value of 1.0. The * indicates a significant difference (p ≤ 0.05) from NS cells by one-way ANOVA followed by Tukey's multiple comparisons. Cell morphology is shown in microscopic images at 200x magnification; scale bar, 200 μm. **D, upper left panel**, Cell proliferation was assayed at the indicated time points. Fluorescent units are presented as the mean ± SD, n = 3; * indicates a significant difference (p ≤ 0.05) from NS cells by one-way ANOVA followed by Tukey's multiple comparisons. **D, upper right panel,** Caspase 3 (CASP3) protein levels were detected by immunoblot. Densitometric units of CASP3 were normalized to ACTB. Values are the mean ± SD, n = 3, relative to M. The * indicates a significant difference (p ≤ 0.05) from NS cells by one-way ANOVA followed by Tukey's multiple comparisons. **D, lower left panel**, CASP3 activity was measured. Fluorescence units are presented as the mean ± SD, n = 3. The * indicates a significant difference (p ≤ 0.05) from NS cells by one-way ANOVA followed by Tukey's multiple comparisons. **D, lower right panel,** Cleaved poly-ADP ribose polymerase (CLVD PARP) protein levels were detected by immunoblot. Staurosporine (STS; 1 μM) treatment served as positive control and was run on same blot. Densitometric units of cleaved PARP were normalized to ACTB and the values are presented as the mean ± SD, n = 3. Densitometric units obtained in the experimental samples were no different from background. Data were analyzed by one-way ANOVA followed by Tukey's multiple comparisons. **E,** Cell migration was determined. Microscopic images of migrating cells after 22 h at 200x; scale bar, 200 μm. **E, upper right panel,** Migrating cells were expressed as a percentage of M. Values are presented as the mean ± SD, n = 5. The * indicates a significant difference (p ≤ 0.05) from NS cells by one-way ANOVA followed by Tukey's multiple comparisons. **E, lower right panel**, qPCR analysis of CYP1B1 RNA levels. Values are presented as the mean ± SD, n = 3, relative to M, set at a value of 1.0. The * indicates a significant difference (p ≤ 0.05) from NS cells by one-way ANOVA followed by Tukey's multiple comparisons. **F,** Cell invasion was assessed using a Transwell matrigel assay. Microscopic images of invading cells after 22 h at 200x; scale bar, 200 μm. **F, upper right panel,** Invading cells were expressed as a percentage of M. Values are presented as the mean ± SD, n = 5. The * indicates a significant difference (p ≤ 0.05) from NS cells by one-way ANOVA followed by Tukey's multiple comparisons. **F, lower right panel,** qPCR analysis of CYP1B1 RNA levels. Values are presented as the mean ± SD, n = 3, relative to M, set at a value of 1.0. The * indicates a significant difference (p ≤ 0.05) from NS cells by one-way ANOVA followed by Tukey's multiple comparisons.

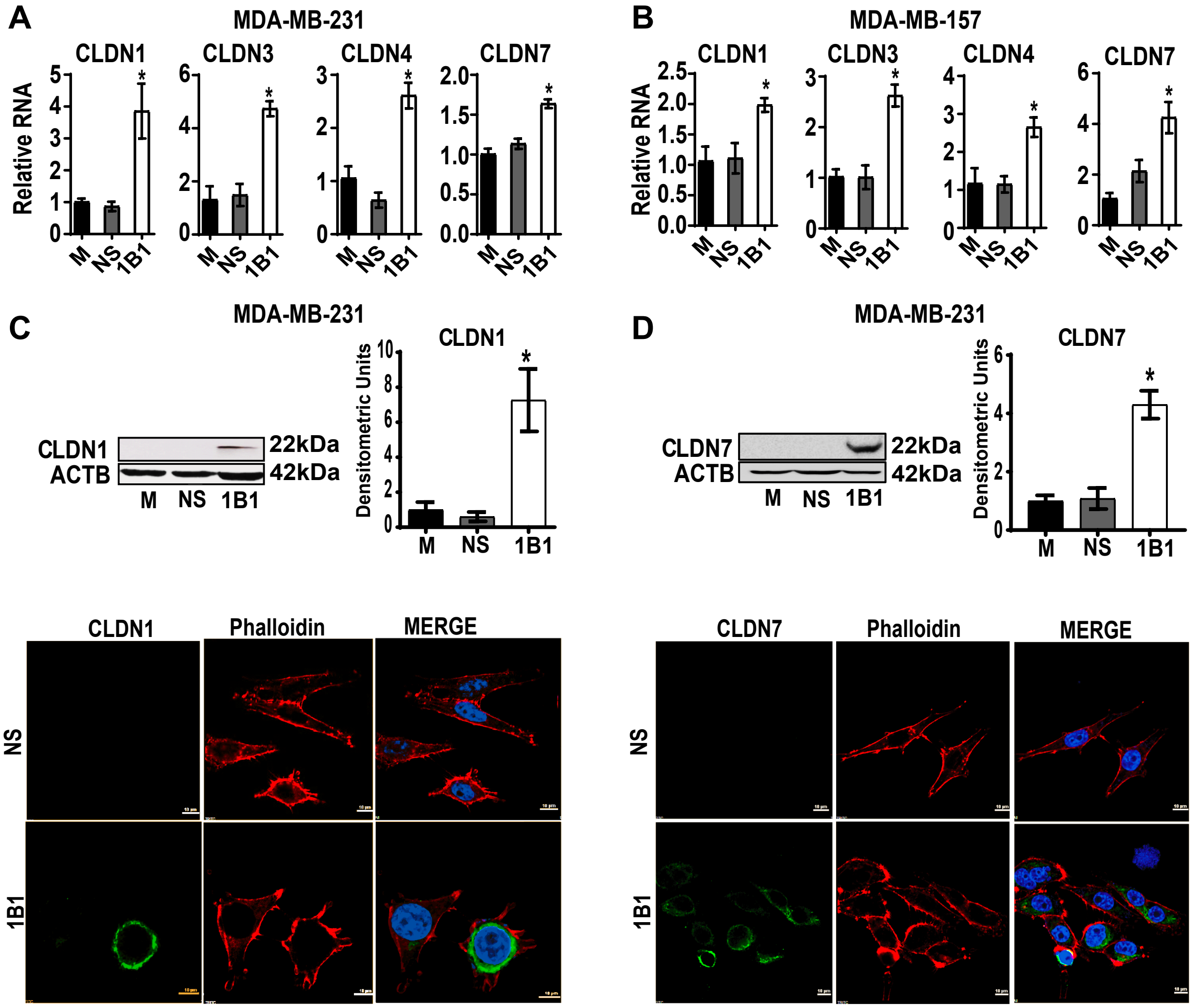

Supplementary Figure S2. CYP1B1 suppression is associated with increased claudin expression in TNBC cells. **A,** MDA-MB-231 cells, M, NS, 1B1 (CYP1B1 siRNA). qPCR analysis of CLDN1, CLDN3, CLDN4, and CLDN7 RNAs. Values are presented as the mean ± SD, n = 3, relative to M, set at a value of 1.0. The * indicates a significant difference ($p \leq 0.05$) from NS cells by one-way ANOVA followed by Tukey's multiple comparisons. **B,** MDA-MB-157, M, NS or 1B1. qPCR analysis of CLDN1, CLDN3, CLDN4, and CLDN7 RNAs. Values are presented as the mean ± SD, n = 3, relative to M, set at a value of 1.0. The * indicates a significant difference ($p \leq 0.05$) from NS cells by one-way ANOVA followed by Tukey's multiple comparisons. **C,** CLDN1 protein levels. Densitometric units of CLDN1 were normalized to ACTB. Values are presented as the mean ± SD, n = 3, relative to M, set at a value of 1.0. The * indicates a significant difference ($p \leq 0.05$) from NS cells by one-way ANOVA followed by Tukey's multiple comparisons. **C, lower panel,** Confocal microscopic images of NS and 1B1 transfected cells; CLDN1 (green) immunofluorescent staining. Nuclei were visualized with DAPI (blue) and F-actin stained with phalloidin (red). Representative confocal microscopic images at 600x magnification, scale bar, 10 μm. **D**, CLDN7 protein levels. Densitometric units of CLDN7 were normalized to ACTB. Values are presented as the mean ± SD, n = 3, relative to M, set at a value of 1.0. The * indicates a significant difference ($p \leq 0.05$) from NS cells by one-way ANOVA followed by Tukey's multiple comparisons. **D, lower panel**, Confocal microscopic images of NS and 1B1 transfected cells; CLDN7 (green) immunofluorescent staining. Nuclei were visualized with DAPI (blue) and F-actin stained with phalloidin (red). Representative confocal microscopic images at 600x magnification, scale bar, 10 μm.

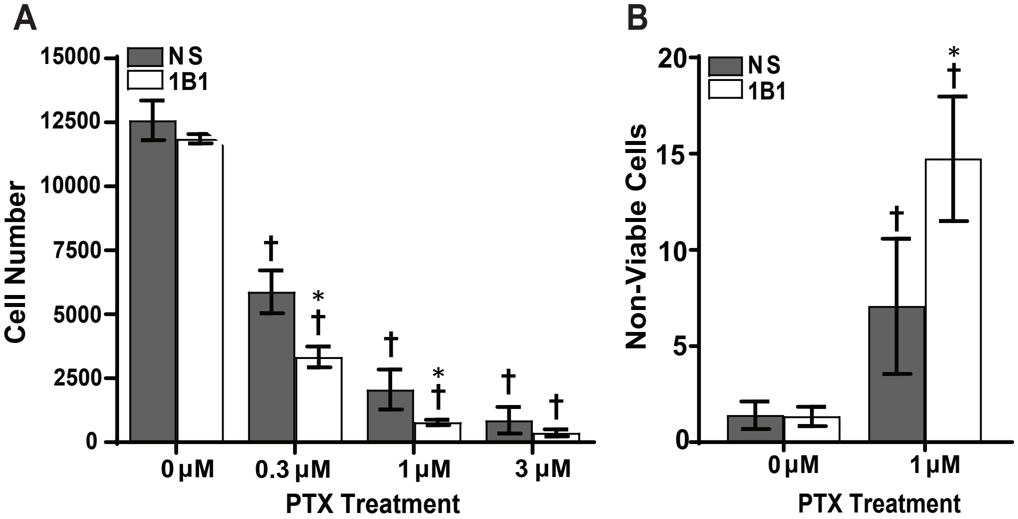

Supplementary Figure S3. Suppression of CYP1B1 sensitizes MDA-MB-231 cells to paclitaxel (PTX). MDA-MB-231 cells were transfected with CYP1B1 siRNA (1B1) or non-specific siRNA (NS). **A,** Cell proliferation was assayed at the indicated time points. NS and 1B1 cells were treated with increasing concentrations of PTX (0.3, 1, or 3 µM, 72 h). Values are presented as the mean ± SD, n = 4. The * indicates a significant difference between NS and 1B1 cells treated with same dose of PTX; the † indicates a significant difference from 1B1 cells treated with vehicle; $p \leq 0.05$ using two-way ANOVA followed by Tukey's multiple comparisons. **B,** Cell viability was assessed using a trypan blue exclusion assay. NS or 1B1 cells were treated with vehicle or 1 µM PTX (72 h). Values are presented as the mean ± SD, n = 3. The * indicates a significant difference ($p \leq 0.05$) between the NS cells and 1B1 cells within the same concentration of drug, and the † indicates a significant difference from vehicle within the same cell line by two-way ANOVA followed by Tukey's multiple comparisons.

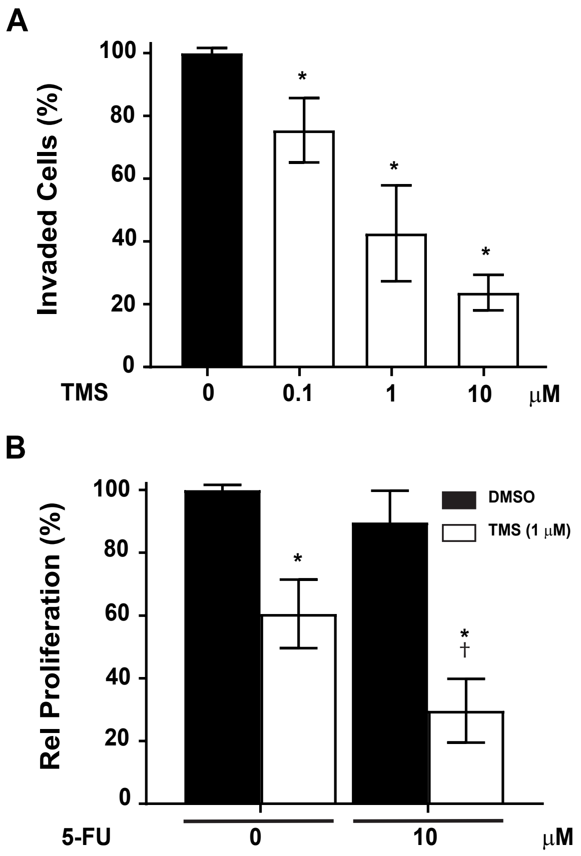

Supplementary Figure S4. Effects of 2,3',4,5'-tetramethoxystilbene (TMS), a CYP1B1 inhibitor, on invasion and cell proliferation. **A,** The CYP1B1 inhibitor, TMS, decreased cellular invasion in a dose-dependent manner. Cell invasion was assessed as invasion through Martigel with or without exposure to the indicated concentrations of TMS for 28 h (Please note difference in treatment time compared to Figure 4). Invading cells were expressed as a percentage of untreated MDA-MB-231 cells. Values are presented as the mean ± SD, n = 5. The * indicates a significant difference ($p \leq 0.05$) from 0 μM TMS by one-way ANOVA followed by Tukey's multiple comparisons. **B,** TMS sensitized MDA-MB-231 cells to 5-FU. MDA-MB-231 cells were treated with DMSO, TMS, and/or 5-FU, as indicated for 48 h, before cell proliferation was measured. Values are presented as the mean ± SD, n = 5. The * indicates a significant difference ($p \leq 0.05$) from the TMS control groups (DMSO) in the 5-FU treatment groups and the † indicates a significant difference from the TMS only treatment group using two-way ANOVA followed by Tukey's multiple comparisons.

Table S1. Primer sequences used in real-time qPCR

	Primers 5'-3'	
	Forward	Reverse Complement
ACTB	TGGTGGGCATGGGTCAGAAG	GTCCCGGCCAGCCAGGTCCAG
CYP1B1	AGCCCAACCTGCCCTATGTCC	CACCGCCTTTTGCCCACTG
SNAI1	CTCGGACCTTCTCCCGAATG	AAAGTCCTGTGGGGCTGATG
SNAI2	CAACGCCTCCAAAAAGCCAA	ACTCACTCGCCCCAAAGATG
FN1	CCACAGCTATTCCTGCACCA	CTGAGCTGGTCTGCTTGTCA
ZEB1	CAGGTGTAAGCGCAGAAA	TGGTCTGTTGGCAGGTCATC
ZEB2	ATGCGAACTGCCATCTGATCC	GCTTCTGGCCCCATAGTGTC
VIM	GGACCAGCTAACCAACGACA	AAGGTCAAGACGTGCCAGAG
DSP	GAGACCGAGATCAACATCACCA	GCTCTCCTCTGTTCGCATCT
KRT8	CAGGGTAGCACTGGGAACAG	TTTGTAGCTGGAGGCATGGG
KRT16	TCCTATTCTTCCCGCGAGGT	TTATTAGCCCACCACCAGCAG
LAMA1	GGAGCTGGAGTTTGACACGA	GAGCATGGAGCTGGAAGGTT
CDH1	AGCTGTTCATACCCTTGTCCC	TGCTGTTCTTCACGTGCTCA
CDH3	GACCGTGAGGATGAGCAGTT	CAGATGGTGATCTGACGGGG
CLDN1	TGAGGATGGCTGTCATTGGG	AAAGTAGGGCACCTCCCAGA
CLDN3	GTCTAAGGGACAGACGCAGG	TCACAGTCCATGCAGGTTGG
CLDN4	CCAACTGCCTGGAGGATGAA	TTGGCGGAGTAAGGCTTGTC
CLDN7	TGGCCATCAGATTGTCACAGA	TGCACCTCCCAGGATGACTA
